# Expulsion and Extraction of the Placenta before the Child

**Published:** 1846-03

**Authors:** James Y. Simpson


*On the Expulsion and Extraction of the Placenta before the Child.* By James Y. Simpson, M. D., F. R. S. E.—All writers on Midwifery concur in the opinion that there is no circumstance more trying to the attendant, or more fatal to the parturient woman than the recurrence of hemorrhage from the presentation of the placenta. So high, indeed, is the mortality arising from this complication, that one woman in three perishes from it, a fatality which is as great as that of cholera, and more so than that of the dangerous capital operations. Such being the case, any attempt, as in the memoir before us, to diminish the fearful risk attendant upon placenta prævia, is, in the words of the author, "at least entitled to the consideration of the obstetric profession, even should it fail to secure their concurrence and convictton."

The principles of treatment in cases of placental presentation hitherto pursued may be reduced to two:—that which has been supposed to be chiefly applicable in *partial* presentation, namely, rupture of the membranes; and that which meets with more general approbation, and has been generally considered indispensable in severe "unavoidable hemorrhage," namely, turning. To these Dr. Simpson proposes to add a third—the *complete separation*, and *extraction*, if *necessary*, of the *placenta before the child.* He has been induced to offer this suggestion, from the circumstance, on one hand, that the most serious hemorrhage always occurs when the placenta is only partially detached; and on the other hand from the fact which he establishes upon the evidence of an extensive and well-arranged table of cases, "that when the placenta, in unavoidable hemorrhage, is once *completely* separated from its connexions with the interior of the uterus, the accompanying flooding in general entirely ceases, or becomes moderate in quantity." It virtually appears, from the examination of the table alluded to, that in 5 only of 111 cases in which the extraction of the placenta preceded the expulsion of the child, did the hemorrhage continue so profuse as to be considered alarming by the attendant.

Dr. Simpson displays the value of his new mode of treatment by a comparison of the mortality under it, and that which occurs under the usual method. It has already been stated that in unavoidable hemorrhage treated by turning the deaths are as 1 to 3. Among the 140 cases tabulated by Dr. S. 10 were fatal or 1 in 14. These figures, therefore, show a striking preponderance in favor of the new plan, but favorable as it is under this point of view, it becomes still more so by an investigation of the circumstances attending the death of the 10 fatal cases. Four of these died several days subsequently to delivery, so that they obviously did not die directly from the effects of hemorrhage; and in three others, one died from the consequences of her husband's brutality, and the two remaining from causes unconnected with the placenta, so that, as Dr. S. observes, in 141 cases the deaths are reduced to 3, or 1 in 47.

In the conclusion of this interesting paper the author draws the following deductions:

1. That the *complete* separation and expulsion of the placenta before the child, in cases of unavoidable hemorrhage, is not so rare an occurrence as accoucheurs appear generally to believe.

2. It is not by any means so serious and dangerous a complication as might *à priori* be expected.

3. In nineteen out of twenty cases in which it has happened, the attendant hemorrhage has been either at once altogether arrested, or it has become so much diminished as not to be afterwards alarming.

4. The presence or absence of flooding after the complete separation of the placenta, does not seem in any degree to be regulated by the duration of time intervening between the detachment of the placenta and the birth of the child.

5. In 10 only out of 141 cases, the mother died after the complete expulsion or extraction of the placenta before the child.

6. In 7 of these 10 casualties, the death of the mother seemed to have no connexion with the complete detachment of the placenta, or with the results arising directly from it, and if we do admit the three remaining cases, which are doubtful, they would still only constitute a mortality of 1 in 47 cases.

7. On the other hand, under the present established rules of practice, the deaths in placental presentation are as high as 1 in 3.—*Lond. and Edin. Jour.*—*Ranking's Abstract.*

